# Single-Particle Optical Imaging for Ultrasensitive Bioanalysis

**DOI:** 10.3390/bios12121105

**Published:** 2022-12-01

**Authors:** Yujie Liu, Binxiao Li, Baohong Liu, Kun Zhang

**Affiliations:** 1 Shanghai Institute of Pediatric Research, Shanghai Key Laboratory of Pediatric Gastroenterology and Nutrition, Xin Hua Hospital, Shanghai Jiao Tong University School of Medicine, Shanghai 200092, China; 2 Department of Chemistry, Shanghai Stomatological Hospital, State Key Lab of Molecular Engineering of Polymers, Institutes of Biomedical Sciences, Fudan University, Shanghai 200438, China

**Keywords:** single-particle imaging, optical imaging, ultrasensitive analysis, nanoparticle labels

## Abstract

The quantitative detection of critical biomolecules and in particular low-abundance biomarkers in biofluids is crucial for early-stage diagnosis and management but remains a challenge largely owing to the insufficient sensitivity of existing ensemble-sensing methods. The single-particle imaging technique has emerged as an important tool to analyze ultralow-abundance biomolecules by engineering and exploiting the distinct physical and chemical property of individual luminescent particles. In this review, we focus and survey the latest advances in single-particle optical imaging (OSPI) for ultrasensitive bioanalysis pertaining to basic biological studies and clinical applications. We first introduce state-of-the-art OSPI techniques, including fluorescence, surface-enhanced Raman scattering, electrochemiluminescence, and dark-field scattering, with emphasis on the contributions of various metal and nonmetal nano-labels to the improvement of the signal-to-noise ratio. During the discussion of individual techniques, we also highlight their applications in spatial–temporal measurement of key biomarkers such as proteins, nucleic acids and extracellular vesicles with single-entity sensitivity. To that end, we discuss the current challenges and prospective trends of single-particle optical-imaging-based bioanalysis.

## 1. Introduction

Despite tremendous progress in modern biomedical science, many major malignancies, including but not limited to infectious diseases, neurodegenerative diseases, and cancer, continue to threaten human health [1,2,3]. For example, incidence rates of malignant brain tumors increased by 0.7% and 0.5% per year from 2008 to 2017 among children and adolescents in the United States [4], and such an increasing trend was also found in China and West Europe [5,6]. Early detection is crucial to reduce cancer mortality; the average five-year survival at early stage is 91% but drops to 26% at advanced stage [3]. Currently, cancer is diagnosed by clinical presentation and imaging findings with final confirmation by tissue biopsy. Despite being the gold standard, the single-lesion tumor biopsy has several limitations such as invasiveness, potential patient morbidity, procedural costs, and inability to capture cancer heterogeneity [7,8,9]. Existing clinical imaging modalities suffer from either low sensitivity (e.g., magnetic resonance imaging) or insufficient specificity (e.g., low dose computed tomography), and in some cases, may arouse concerns regarding potential harm from radiation exposure [10,11,12]. As such, these unmet clinical needs provide motivation to develop novel non-invasive tools for early detection and tracking of malignancies. Recent multi-omics studies have offered solid and conclusive evidence that molecular alterations of the genome, transcriptome, proteome and metabolome precede symptom onset [13,14,15,16]. Moreover, these molecular substances derived from abnormal cells and other microenvironment factors, such as immune cells, can be actively released into the extracellular space and further into body fluids (e.g., blood) in soluble, membrane-bound or -encapsulated forms, proving rich clues for disease occurrence and evolution [17,18,19,20]. Analysis of these circulating species, therefore, holds great potential as a non-invasive ‘liquid biopsy’ approach to enable early diagnosis, dynamic monitoring and accurate predication of therapeutic response in the context of precision medicine.

To date, a large variety of biochemical methodologies, such as polymerase chain reaction (PCR), enzyme-linked immunosorbent assay (ELISA), colorimetric and luminescent assays, have been established for the qualitative and quantitative analysis of circulating biomolecules [21,22,23,24,25,26,27,28]. Generally, the average response of a large amount of analytes is measured as an assay signal. Although widely used for decades, these ensemble methods may suffer from inadequate sensitivity to detect low analyte concentrations. For example, classical ELISA approaches show the limits of detection at concentrations above 10^−12^ M, whereas the circulating levels of many protein biomarkers associated with cancer, neurological disorders, and the early stages of infection frequently fall in the femtomolar (10^−15^ M) range and below [29,30,31,32]. The weak detection signal combined with the intense background interference leads to the unsatisfied assay performance of these ensemble approaches at low analyte concentrations. Therefore, great efforts have been devoted to the enhancement of detection signals by preconcentration, and more commonly, introducing a signal amplification element (e.g., enzyme) into the assay system [33,34,35]. However, this may in turn complicate the assay procedure and result in an increased background along with improved sensitivity. In this context, alternative strategies capable of creasing the signal-to-noise ratio (SNR), thereby enhancing the analytical sensitivity, should be pursued.

The last decades have witnessed rapid evolution of bioanalytical studies from the ensemble-averaged measurements to so-called single-entity studies [36,37,38]. Compared to the former, the single-entity approaches offer major benefits, not only in the field of fundamental biomedicine science to enable examination of heterogeneity across individual targets within a population [39,40], but also as promising tools for medical diagnostics by detecting single targets, including single cells, single vesicles, and single molecules, which, at first sight, represent the ultimate sensitivity [41,42,43,44]. Although the ability to detect a single cell or biomolecule does not mean the most sensitive bioanalytical approach, the signal-readout mode of single-entity detection is distinct for the reduction of background signals when significantly decreasing the detection volume. Since the signal arising from a single target analyte can be reliably distinguished from the background noise, the assay is independent of background fluctuations, which makes the assay more robust and indirectly results in lower detection limits [45]. Among various techniques [46,47,48], optical spectroscopic/microscopic methods were first employed [49] and are still the main analytical tools allowing for single-entity investigation. In practical analysis, two measurement modes can be adopted: in label-free analysis, the adsorption/desorption of a single target on the detection zone leads to an optical signal response that can be directly measured [50]. Alternatively, individual entities of interest are labeled with signal reporters, the optical response of which can be detected to indicate the status of the target analytes [51]. Fluorescent dyes with a high emission quantum yield are the most common elements used for optical labeling owing to their small sizes and the well-established bioconjugation chemistry [52,53]. The imaging and analysis of individual dye-labeled cells, proteins, and nucleic acids have greatly deepened our understanding of fundamental life processes [54,55,56]. However, applying these strategies for in vitro quantification of circulating biomarkers in real-world clinical settings remains difficult. The limited brightness of single fluorescent dyes means that a microscopy imaging system integrating a high-cost advanced photon collection and detection module (e.g., electron-multiplying charge-coupled device and objective lens with a high numerical aperture) is required to excite and record the luminescent signals of individual fluorophores. In the meantime, an optimized assay condition is desired to avoid quenching, photobleaching and photodegradation. It is almost impossible for the majority of medical testing laboratories and in particular those in resource-limited settings to meet such requirements.

Nanoparticles (NPs) may provide a solution for the above predicament. Due to their high specific surface area, tunable physicochemical property, and ease of functionality, NPs have attracted particular attention as labeling materials in affinity-based bioassays [57]. Plasmonic metal NPs, such as Au NPs and Ag NPs, strongly absorb and scatter light such that direct observation of individual NPs under standard dark-field microscopy (DFM) is feasible [58]. Apart from providing signals by their intrinsic ‘photo emission’, NPs with plasmonic properties can also be used to enhance the ‘emission’ of molecules located near their surfaces, giving birth to the field of plasmon-enhanced spectroscopy [59]. Compared to organic molecular fluorophores, NPs such as quantum dots (QDs) and polymer dots (PDs) are much brighter and more photostable, which are important features for single-entity analysis by fluorescence imaging. On the other hand, NPs operating in the near-infrared region or with photo-upconversion properties (excitation by near-infrared light, anti-Stokes emission at shorter wavelength) are resistant to background interference from biological samples [60]. In addition to using photoluminescent NPs as labeling elements, the background-free detection can also be achieved by implementing nanolabels with other emission ways such as electrochemiluminescence (ECL), a transduction process involving the generation of species at electrode surfaces that then undergo electron-transfer reactions to form excited states that emit light [61]. Since excitation light is not required in the measurement, ECL analysis provides a completely dark background and allows optical observation without interference from autofluorescence and scattering [62]. Due to the above merits, the optical imaging of single luminescent particles (OSPI) after labeling them to the individual analytes of interest provides a better alternative to classical single-molecule imaging analysis of low analyte concentrations. 

This review summarizes the technical advances of OSPI-based bioanalysis in the most recent five years. Notably, we focus on the label-based studies in this review, although several novel label-free optical imaging methods, such as plasmonic scattering microscopy and interferometric scattering microscopy [50,63], have also gained great progress during this period, with an ability to inspect individual analyte targets with high spatial and temporal resolution. Readers interested in label-free techniques can refer to the outstanding papers published recently [63,64,65,66,67]. According to the readout modality, we pay particular attention to four types of OSPI approaches, which include fluorescence, ECL, surface-enhanced Raman scattering (SERS), and dark-field scattering. For each technique, we first introduce basic concepts that are important in understanding of the optical phenomenon. Then, we elaborate on major progress in analyzing low-abundance biomolecules by the corresponding OSPI techniques. Finally, we discuss the existing limitations and potential future directions in OSPI-based bioanalyses. We expect that this review will offer valuable insights into OSPI-based techniques and will be helpful for those engaged in developing ultrasensitive reliable bioanalytical approaches to quantify low-concentration circulating biomarker candidates, thereby spurring further interest in this research frontier.

## 2. Ultrasensitive Bioanalysis by Different OSPI Techniques

### 2.1. Single-Particle Fluorescence Imaging

Fluorescence is the emission of light by a photo-excited fluorophore that occurs within nanoseconds. Fluorescence imaging is perhaps the most popular optical imaging modality that has long been employed in various biological and medical studies, initially for observing molecular populations at diffraction-limited resolution and now capable of probing single molecule dynamics at nanometer and millisecond spatial–temporal resolutions [68,69]. Single-particle fluorescent probes, such as dye-labeled NPs, QDs, and upconversion NPs (UCNPs), have additional merits of biocompatibility and long-term imaging ability. Taking the dye-labeled AuNPs as an example, the rational assembly of hundreds of dye molecules on the surface of individual AuNPs contributes to the long-term imaging application of the probes by providing a largely elevated overall luminescent intensity of single particles under mild excitation conditions [70,71]. Moreover, it has been reported that AuNPs could enhance fluorescence and photostability, as well as biocompatibility [72,73]. In terms of QDs, the photostability of them is superior to various dyes, making QDs excellent candidates for long-term single-particle imaging [74]. In this context, a variety of functional QDs have been developed and applied for long-term imaging and tracing of analytes, including cell-derived microvesicles [75], intracellular pH [76], proteins [77], metal ions [78], and so on. As discussed above, one of the prominent advantages of UCNPs is manifested in low autofluorescence, which makes them suitable for long-term high-resolution imaging and tracking of biomolecules [79].

**Figure 1:**
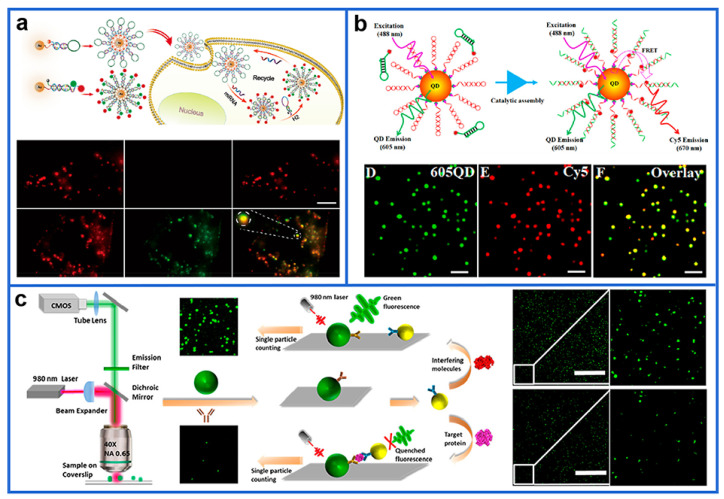
(**a**) Schematic of miRNA detection by the dual-color fluorescence imaging. (**b**) Principle of the catalytic assembly of the QD nanosensor directed by toehold-mediated strand displacement cascade. (**c**) Schematic diagram of the light path for the optical microscopic imaging of UCNPs and the principle of digital immunosorbent assay by single-particle enumeration. Reprinted with permission from [84,95,103]. Copyright 2020, 2019, and 2018, American Chemical Society.

Owing to the small size and easy of bioconjugation, organic dyes are extensively used as fluorescent imaging labels in biological laboratories [80,81,82]. For example, our group reported a series of single-molecule fluorescence assays for high-sensitivity and high-specificity detection of biomolecules [83,84,85,86]. In one of our recent works, a single-molecule fluorescence imaging assay was proposed for the ultrasensitive dual-plex detection of miRNAs using the S9.6 antibody to capture the dye-labeled DNA–RNA hybrids [83]. Considering the weak emission of organic dyes, a dual-color fluorescent nanoprobe was constructed in our laboratory by using Au NPs as scaffolds to immobilize many dye-labeled nucleic acid probes, which was then applied to detect the low-abundance miRNAs in single cells (Figure 1a) [84]. In the assay, the self-assembly process between target miRNA and the fluorescent probes improved the sensing accuracy, and the two-color imaging mode effectively diminished the false-positive signals caused by chemical interference. Unsurprisingly, the monitoring of miRNAs at femtomolar levels was achieved through a combination of single-molecule fluorescence imaging and enzyme-free signal amplification. The proposed method was further used for high-resolution three-dimensional analysis of key miRNAs in single migrating cells [85,86]. Our results clearly showed the differential expression and intracellular locations of miRNAs in different cell lines. 

Compared with organic molecular fluorophores, QDs have several unique advantages: (1) Photostability. The single QDs is 20 times as bright, 100 times as stable against photobleaching of Rhodamine [87] and even more stable than that of AlexaFluor 488 [88], making it an ideal material for single molecule/particle imaging and tracking applications. (2) Fluorescent activity. QDs exhibit strong fluorescent activity due to their high quantum yield (0.1–0.8 under visible, 0.2–0.7 under near-infrared) [89] and high molar extinction coefficient in the order of 5.5 × 10^6^ M^−1^ cm^−1^ [90]. (3) Fluorescent lifetime. The fluorescent lifetime of QDs (10–20 ns or greater) is longer than that of dye molecules (<5 ns) [91], resulting in a fluorescent-signal-diminished background interference. (4) Biocompatibility. QDs can be biofunctionalized with diverse chemical or biological ligands such as polyethylene glycol, enhancing their biocompatibility. Accordingly, QDs can be used for biological in vivo labeling and detection. (5) Stokes shift. The large Stokes shift (e.g., stokes shifts of semiconductor QDs can be as large as 300–400 nm) [91] reduces the overlap of emission spectrum and excitation spectrum, thus facilitating the detection of fluorescence spectrum signals. (6) Size-tunable emission. The emission spectrum of QDs can be tuned by changing their size and composition, in which one light source can be used to excite multiple colors of fluorescence emission. (7) Wide excitation spectrum and narrow emission spectrum. Using the same excitation light source can realize synchronous detection of QDs with different sizes; thus, it can be used for multi-color labeling and greatly promotes the application in fluorescent labeling [92]. In addition, the narrow and symmetrical fluorescence emission peaks of QDs avoid the spectral overlap. Zhang’ group established many single QD-based fluorescence imaging methods for biomarker quantification [93,94]. Using QDs as energy donors, and integrating fluorescence resonance energy transfer (FRET) with single-particle counting, they constructed a series of biosensors with high spatiotemporal resolution and sensitivity [95,96,97,98]. Figure 1b shows a single QD-based FRET nanosensor [95]. In the presence of target miRNAs, Cy5-labeled signal probes and biotinylated capture probes were hybridized to form capture probe/signal probe duplexes. These duplexes can be assembled on the 605QD surface by high affinity interaction, resulting in significant FRET between 605QD and Cy5 in close proximity, allowing for easy and sensitive quantification of the FRET signal by single particle counting for high-quality imaging analysis of miRNAs. In contrast, no FRET occurs without target miRNA. This single QD-based FRET nanosensor can with femtomolar sensitivity allow for the detection of circulating miRNAs in clinical serum samples and imaging target miRNA in living cells. Furthermore, using QDs (QS585) as fluorescent labels, Liu et al. [99] developed a single-molecule counting-based ultrasensitive assay for facile and direct detection of DNA ethyltransferases. Combined with the superior luminous intensity of QS585 and the ultrahigh sensitivity of single-molecule counting, the proposed assay displays a lower detection limit of 0.0005 U/mL. Furthermore, single QD sensitivity and high SNR have been realized [100]. Here, a nearly 3000-fold signal enhancement was achieved through multiplicative effects of enhanced excitation, highly directional extraction, quantum efficiency improvement, and blinking suppression. Consequently, the proposed strategy was applied for highly specific analysis of miRNA to provide digital resolution of individual target molecules, resulting in a low detection limit (~10 aM), single base-pair mismatch selectivity, and a high dynamic range (nine orders of magnitude). In the meantime, that imaging system was capable of recording the dynamic trajectory of single QDs, which could discriminate a single base difference in a target miRNA molecule in 10 min.

Upconversion nanoparticles (UCNPs) under near-infrared excitation are a class of luminescent materials that could resist background interference from biological samples, providing an excellent candidate for the labels of single-particle biosensing [101,102]. Based on the FRET between Au NPs (as accepter) and UCNPs (as donors), a simple yet sensitive sandwich-immunotype single particle counting assay for the visual quantification of prostate-specific antigen (PSA) was constructed (Figure 1c) [103]. Target PSA triggered the specific immunoreaction and brought the donor and acceptor into close proximity, resulting in quenched luminescence. Through statistical counting of the target-dependent fluorescent particles on the glass slide surface, the proposed method allowed for highly sensitive (detection limit: 1.0 pM) detection of PSA in the solution. Notably, a lower detection limit (2.3 pM) was also achieved in serum sample assays, providing assistance for selective detection of cancer biomarkers in clinical diagnosis. Using a similar mechanism, a sensitive single-particle aptasensor was proposed for the detection of aflatoxin B_1_ in peanut samples [104]. Recently, Gorris et al. introduced a single-molecule upconversion-linked immunosorbent assay to detect PSA, which could detect attomolar protein concentrations over conventional immunoassays [105]. Specifically, the labeling system consisting of biotin-PSA antibody and streptavidin-UCNPs ensured that the antibody has better access to PSA bound to the surface of the microtiter plate. The strong affinity streptavidin–biotin pairs can compensate for the sterically constrained access of the UCNP nanotags to the biotinylated antibody on the microtiter plate surface. Consequently, the digital detection of PSA provided a >16-fold lower detection limit compared with the respective analogue detection, and the advantages of the digital mode became more distinctive when the level of nonspecific binding was low.

In response to growing demands for reliability and practicality of imaging sensing, organic/inorganic nanohybrids were developed [106]. Through rational design of the structural parameters regarding composition, morphology and size, a variety of optical properties and biological functions can be achieved [107]. Recently, Habuchi et al. [108] obtained a series of shortwave infrared-emitting PDs with different sizes by nano-precipitating a saturated solution of conjugated polymers in tetrahydrofuran and changing the mixing volume ratio of the organic to aqueous phase. Due to its high fluorescence saturation intensity and large absorption cross section, these PDs exhibited brighter fluorescence compared to commonly used fluorescence materials and achieved millimeter-deep single-particle fluorescence imaging by the tissue phantom. Furthermore, a novel time-gated single-particle imaging modality was developed due to its characteristic spectral properties. This work represents an important step toward millimeter-deep imaging of the entire sample tissue at the single-particle level. 

The single-molecule biosensors based on fluorescence imaging are quite simple and sensitive, holding great potential for further application in clinical diagnosis and monitoring. However, great efforts should be put into expanding their reliability and utility. First, improving the SNR of the fluorescent tags will contribute to improving the sensitivity and accuracy of the sensors. Second, new fluorescent labeling strategies will be beneficial to keeping high and stable performance of the biosensors. Third, more economical and automated instruments are needed to be developed.

### 2.2. Single-Particle ECL Imaging

Apart from using photoluminescent NPs as labeling elements, the background-free detection can also be achieved by implementing nanolabels with other emission ways. For example, ECL is the phenomenon of light emission triggered by electrically excited chemicals [61]. Due to its nearly zero-background signal and excellent controllability, ECL has been used for the visualization of individual immobilized objects and entities with spatiotemporal resolution, such as single cells [109,110], single NPs [111,112], single microbeads [113,114], and single-molecule electrochemical reaction [115,116]. Shortly after its debut, ECL has been implemented as a powerful imaging technique to visualize and detect biomarkers at the single-molecule level.

**Figure 2:**
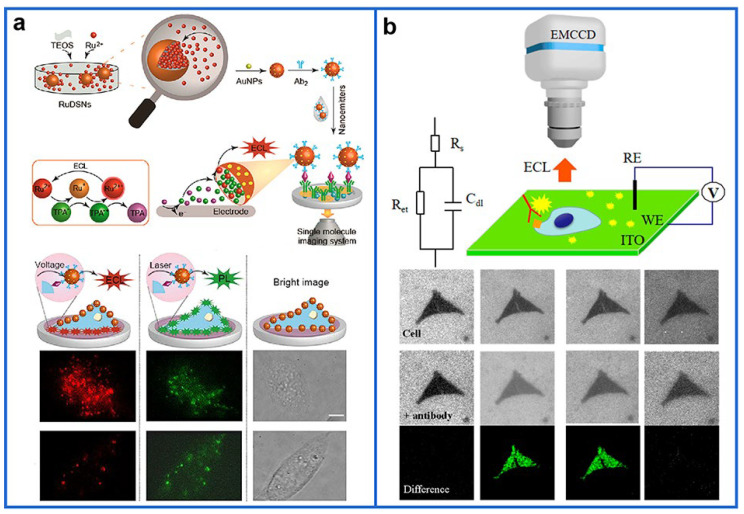
(**a**) Schematic illustration for single-protein molecule imaging by ECL. (**b**) Schematic diagram of the label-free electrochemiluminescence-based capacitance microscopy for imaging of antigens on the cellular plasma membrane. Reprinted with permission from [117,124]. Copyright 2021 and 2019, American Chemical Society.

Exploring novel ECL systems with significantly enhanced luminescence is an important way to achieve ECL detection at the single-molecule level. Our group achieved the first ECL imaging of a single protein on the cell membrane based on the surface confinement of single ECL nanoemitters (Figure 2a) [117]. In that study, Ru(bpy)_3_^2+^-doped silica (RuDSNs)/Au NPs were developed as the ECL nanoprobe, where the ECL emission is confined to the local surface of RuDSNs, which resulted in a significant enhancement in the ECL signal intensity. As a proof of concept, the RuDSNs/Au NPs emitters were then modified with antibodies to achieve spatially resolved imaging of individual proteins on the single cell surface. Compared with PL images, an increase in the SNR of ~17-fold in the ECL images exhibited the improved contrast of single biomolecule imaging. The successful visualization of a single protein at the electrode surface and cellular membrane indicated that the local surface confinement effect enabled spatially resolved imaging of single proteins, which opened up a new field in the biological application using ECL imaging. Subsequently, inspired by the nanoconfinement effect and high reactivity in the metal organic framework (MOF), Li et al. designed the Ru(bpy)_3_^2+^-embedded MOFs (RuMOFs) as ECL nanoemitters [118]. The unique multipole confined space in RuMOFs facilitates the electron/proton transfer and improves the accumulation of intermediate radicals, which permit high-quality imaging of individual ECL events and a stable ECL emission up to 1 h. By labeling individual proteins of living cells with single RuMOFs, this nanosystem achieves real-time ECL monitoring and dynamic mapping of protein molecules, and effectively differentiates the heterogeneity of movement direction and velocity among protein individuals in different regions. This work constitutes a valuable contribution to steer meaningful exploration in this direction of ECL imaging and gain insightful visible information regarding cells or small biomolecules. Moreover, Paolucci et al. [113] discovered an unexpected but highly efficient (signal enhancement, 128%) mechanistic path for ECL generation using an innovative combination of ECL imaging techniques and electrochemical mapping of radical generation. The ECL emissions from single-labeled micromagnetic beads (MB) near the electrode surface (≤1 μm) were mapped, revealing the contribution of an additional pathway to ECL generation. In particular, they coupled streptavidin-coated MB with biotin-functionalized ruthenium-containing antibodies to quantify multiple proteins.

Nevertheless, the toxicity and corrosiveness of the tripropylamine co-reactant limit its application in bioanalysis [119]. In addition, in order to observe the whole basal contact, in the above studies, cells have to be treated with reagents to change the permeability of the cell membrane, which might lead to cell damage. Here, Ju et al. [120] designed a dual intramolecular electron transfer strategy by introducing two tertiary amine groups to the side chain of the polymer unit, which led to a co-reactant-embedded ECL system of PDs for the first time. Encouragingly, the superstructure and intramolecular electron transfer brought an unprecedented ECL emission, making it suitable for in situ ECL microimaging of a membrane protein on single living cells without additional permeable treatment for transporting the co-reactant.

Most current ECL imaging studies use labeling methods to identify target molecules on the electrode surface, which is time-consuming, labor-intensive, and prone to interference. Label-free ECL sensing methods have been paid gradual attention [121,122]. Thus, label-free shadow ECL microscopy was reported [123]. Based on the spatial confinement of the ECL-emitting reactive layer, the single living mitochondria deposited on the electrode surface was imaged. For single protein detection, label-free ECL-based capacitance microscopy was first established by Jiang et al. to visualize the analyte on the electrode surface and even on the plasma membrane of single cells (Figure 2b) [124]. Upon binding of species to the surface or to a cellular membrane, the drop in the local capacitance was derived to induce a relatively larger potential drop, which was utilized to prompt enhanced ECL at the binding position. Using this new detection principle and resultant capacitance microscopy, target proteins at amounts of as low as 1 pg could be visualized. Further application of this approach permitted the direct imaging of protein antigens on single cells through the capacitance change after the formation of the antigen–antibody complex.

Although there has been great progress in ECL imaging, it still remains challenging. Novel ECL nanoprobes and reaction strategies with higher luminous efficiency should be excavated to measure analytes at the single-molecule level. Great efforts to establish novel ECL methods with higher stability and better reproducibility should be paid attention to minimize chemical and electrical interference. It is desirable to develop an economical, reliable, fast, and convenient SP ECL platform to afford large-scale clinical testing.

### 2.3. Single-Particle SERS Imaging

Currently, SERS imaging technology for the visualization of life-related functional molecules has attracted considerable interest due to its wider range of excitation wavelength, high spectral resolution, low biological autofluorescence, and no quenching or photobleaching [125]. In particular, taking advantage of the excitation of the localized surface plasmon resonance on metal NPs, SERS offers an ultra-high sensitivity at the single-molecule level and enables the precise localization and identification of single NPs, making it a reliable method and imaging tool in biological analysis, environmental monitoring, medical diagnosis, etc. [126,127,128].

**Figure 3:**
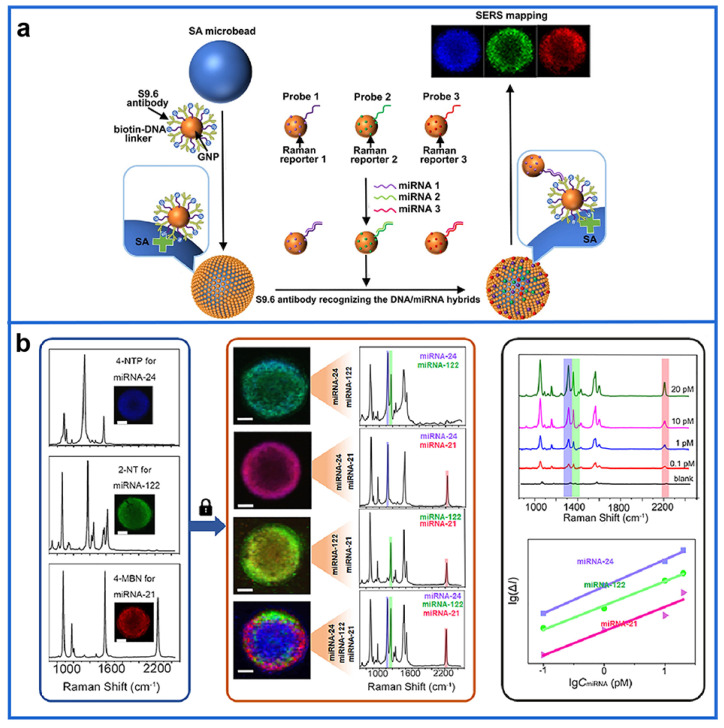
(**a**) Scheme of the multiplexed miRNA assay on a single plasmonic microbead. (**b**) Analytical performance of the proposed methods. Reprinted with permission from [131]. Copyright 2021, American Chemical Society.

Benefiting from the narrow line width of Raman spectra, Trau et al. [129] developed a digital nanopillar SERS platform that enabled real-time single cytokine counting and dynamic tracking of immune toxicities in cancer patients receiving immune checkpoint inhibitor therapy. In this work, the adoption of the novel digital quantification mode in SERS using Au–Ag alloy nanoboxes significantly improved the sensitivity, and the use of digital SERS signals from the single-SERS-active nanoboxes on discrete pillar arrays made the SERS analysis more accurate and reliable. More importantly, that assay could resolve the highly distinctive spectral peaks of different tags, opening new gates to the simultaneous detection of multiple biomolecules. In their other work, a SERS-based single-molecule-resolution digital extracellular vesicle (EV) counting detection chip was developed to create lung-cancer-associated EV molecular profiles [130]. In addition, a multiple miRNA detection method based on SERS mapping on solely a single microbead was developed, as shown in Figure 3 [131]. Specifically, a single microbead covered with a plasmonic layer was employed as a microreactor for the multiplexed miRNA analysis without nucleic acid amplification. On the plasmonic layer, the antibody was adopted as the universal module for binding DNA/miRNA duplexes regardless of the sequence. Meanwhile, other SERS probe-labeled Au NPs were used to identify the given miRNA. The target miRNA would trigger the specific capture of the corresponding SERS probe-labeled Au NPs onto the plasmonic layer, which enormously enhanced the SERS signals. Ultimately, the enhanced SERS signals concentrated on the microbead would be mapped out by a confocal Raman microscope. Intracellular pH is also one of the key factors for understanding various biological processes in biological cells. Several plasmonic NPs have been extensively studied in SERS for pH sensing [132,133]. To determine pH value at the single particle level, Kawata et al. [134] synthesized 18 types of Au and Ag NPs with different morphologies and quantitatively compared their SERS performance efficiency for fast pH sensing. 

SERS imaging realizes the visualization, quantification, and functional research of biological molecules, which is of great significance in the fields of biomedical imaging and clinical diagnosis [135]. However, there exists a long-term gap between laboratory research and practical biomedical applications of SERS imaging. Generally, the authenticity and stability of single-particle SERS imaging signals need to be improved. The interference of nanoprobes on the equilibrium of the biological systems cannot be ignored. It is necessary to combine with big data analysis for quickly obtaining stable and reliable results.

### 2.4. Single-Particle Dark-Field Scattering

DFM has several inherent advantages, including low background, simple optical setup, and ease of operation [136]. Notably, depending on its structure, a single plasmonic metal NP can be thousands to millions of times brighter than a single QDs or fluorescent dye [137,138]. At present, the continuous development, application, and exploration of DFM techniques have permitted direct acquisition of high-quality color photographs and the corresponding scattering spectra from single plasmonic NPs, making it possible for spatial–temporal observation of molecule–particle interactions at the single NP level [139]. According to the signal changes caused by the size, composition, morphology, or microenvironment of the plasmonic nanoparobes, researchers have developed several DFM analysis methods by tracing the structural-change-induced spectral shift, by imaging and counting the surface-bound single NPs, and by monitoring the changes in scattering intensity of single NPs, etc.

Among them, single-particle enumeration is the most direct and easiest method. Our group developed a single-particle enumeration method based on a phosphorylation directed in situ assembly of Au NPs for the ultrasensitive sensing of cellular protein kinase A (PKA) activity [140]. In the presence of adenosine triphosphate, PKA catalyzed phosphorylation of the polypeptide chain substrate to introduce Au NPs as the plasmonic nanolabels. By measuring the variance of the Au NP counts using DFM, the PKA activity can be quantitatively analyzed. A low detection limit of 1.5 × 10^−7^ U/μL was obtained during the analysis because of the controllable immobilization of capture peptides, efficient capture of nanoprobes, and low background of DFM. With this method, we also realized the simple and sensitive quantification of sortase A (SrtA) activity [141]. As far as we are aware, it is the first time establishing a DFM-based SrtA activity counting detection method. A similar method was made by Evans et al. [142], who presented a novel in vitro protein enumeration strategy based on DFM imaging combined with computational analysis. In that work, DFM equipped with customized image acquisition software was employed for acquiring 3D cell images by utilizing highly specific monoclonal antibody-functionalized Au NPs as contrast-generating probes to visualize and enumerate target proteins on the surface of single cells. Owing to the strong scattering signals of Au NPs, the developed algorithm could be utilized to process thousands of acquired images for rapid visualize and enumerate the bound Au NP probes.

**Figure 4:**
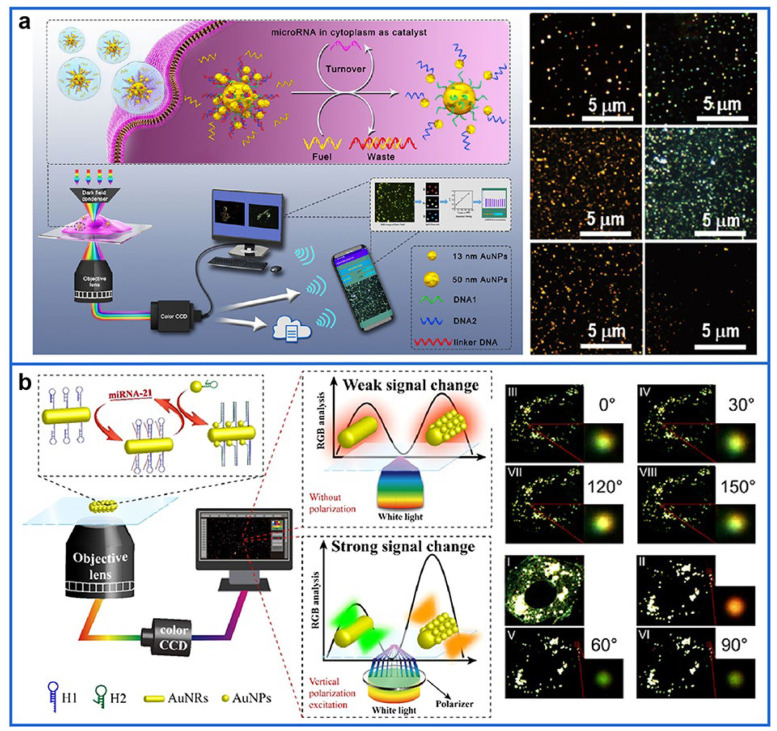
(**a**) Principle of the algorithm-assisted miRNA detection and imaging system. (**b**) Schematic diagram of low-background and high-sensitivity detection of miRNA under vertical polarization excitation. Reprinted with permission from [144,146]. Copyright 2021 and 2020, American Chemical Society.

The scattering spectra and color changes caused by the assembly of plasmonic NPs are also widely applied for the quantification and analysis of target substances. Recently, a massively parallel single particle sensing method based on core-satellite (CS) formation of Au NPs for the detection of cytokine interleukin 6 (IL-6) was introduced [143]. In the presence of IL-6, the localized surface plasmon resonance (LSPR) of Au NPs would change as a result of CS formation, resulting in a change of the observed color. The hue (color) value of thousands of 67 nm Au NPs immobilized on a glass coverslip surface was analyzed by a Matlab code before and after the addition of reporter NPs containing the IL-6 target protein. The method was able to analyze the hue values of thousands of NPs in parallel in less than a minute and circumvent the effect of non-specific adsorption. In addition, a novel algorithm-assisted miRNA detection and imaging system based on the disassembly of plasmonic CS is presented in Figure 4a [144]. The strand displacement amplification was used to amplify color changes in the scattering light, effectively improving the detection sensitivity of the system. The concentration of miRNAs could be acquired quickly and precisely from the DFM images of the probes based on the proposed algorithm without the need for spectrometers, achieving efficient and low-cost detection. Importantly, a smartphone application based on the proposed algorithm was developed, promoting the development of remote diagnosis. Similarly, a doxorubicin-loaded CS nanoprobe for miRNA detection, targeting drug release and therapy evaluation was developed [145]. The plasmonic CS nanoprobe was constructed with uniformly distributional 50 nm (core) and 13 nm (satellites) Au NPs, which were functionally assembled with a specific sequence of DNA and peptides. The constructed CS nanostructure was disassembled in the presence of target miRNA, producing characteristic LSPR signals and releasing doxorubicin. With the increase in the target miRNA concentration ranging from 0.01 to 1000 fM, a distinct blue shift of scattering spectra peak occurred, along with obvious color change from orange to green under DFM, which can be used to detect miRNA at the single-particle level. To allow the DFM analysis in a more automatic, sensitive, objective, and repeatable way, Wang et al. [146] used the vertically polarized excitation of the polarizer to reduce the signal background, realizing a highly sensitive detection and spatial imaging of intracellular miRNA-21. As seen in Figure 4b, target miRNA-21 triggered the CS coupler between Au nanorods (AuNRs) and AuNPs. Normally, the CS assembly presented a similar red color and scattering intensity. However, when the polarization excitation was perpendicular to AuNRs, the lateral light scattering of AuNRs was greatly enhanced and the color of the DFM images obviously changed from green to red due to the coupling between AuNPs and AuNRs along the lateral direction. Subsequently, the red light scattering percentage change of the CS assembly was analyzed to achieve the sensitive detection of miRNA-21, which effectively reduced the strong background signal to improve the detection sensitivity and finally achieved high spatial imaging in living cells.

The light scattering intensity of NPs can be extracted by analyzing the obtained dark field images with the Image-Pro Plus (IPP) open-source software, or by directly reading from the scattering spectrum, and further using the change of scattering intensity to establish an analysis method [147]. In this context, DFM was applied to record the state changes of single-molecular binding and unbinding events that modulate the Brownian particle motion [148]. Using DNA and protein as model biomarkers, the method was validated in buffer and in blood plasma, showing sensitivity to picomolar and nanomolar concentrations. Encouragingly, with its basis in reversible interactions and single-molecule resolution, the presented assay will enable biosensors for continuous biomarker monitoring with high sensitivity, specificity, and accuracy. Recently, NP-enhanced extracellular vesicle immunoassays (NEIs) that are read by DFM found in many research studies and clinical laboratories can be used for the ultrasensitive detection of cancer-associated extracellular vesicle (EV) biomarkers in serum, but these approaches require manual identification of fields of interest, making them prone to operator error and bias [149]. To solve this issue, Hu et al. [150] investigated an NEI workflow permitting automatic capture of DFM images that were then processed with a custom noise-reduction algorithm to reduce artefacts from serum aggregates, particulates and surface scratches introduced during the assay. 

Table 1 summarizes the representative OSPI strategies for the quantitative analysis of biomarkers.

None of these techniques are perfect. Each technique has its own pros and cons. The high sensitivity and spatial–temporal resolution of single molecule fluorescence imaging analysis bring great advantages, but the false-positive events caused by fluorescent impurities and non-specific adsorption, as well as the requirement for high-cost equipment, currently limit its applications and scope. ECL imaging has attracted much attention in single molecule/particle imaging due to its almost zero background signal and controllability. Nevertheless, the light intensity and stability of the ECL emitters need to be improved. Moreover, nontoxic and friendly ECL reaction systems need to be further explored for the real-time imaging of living cells and organisms. SERS imaging shows tolerance to autofluorescence and photobleaching and enables multiplex bioimaging, but the fluctuation of SERS signals and the relatively low throughput of SERS analysis should not be ignored. The low background and simplicity make DFM imaging successful; however, similar to other NPs-based imaging probes, the relatively large size of individual metal NPs means that one should be concerned about the possibly exacerbated non-specific adsorption of imaging probes at the sensing interface.

## 3. Summery and Outlook

This review aims to provide a brief overview of recent technical progress in optical-imaging approaches with single-entity sensitivity that hold potential in future biomedical studies and clinical practice. Although this field is developing rapidly, there are challenges that must be overcome before these techniques can realize their full potential. First, while OSPIs could push the assay sensitivity from low picomolar levels to attomolar levels, one should recognize that such exciting performance is almost always obtained from analysis of an idea sample (e.g., a compound standard in PBS) by a custom-designed optical microscope setup. When they are applied to analyses of biological and clinical samples, the sensitivity would inevitably be compromised to varying degrees due to interference of large numbers of coexisting species in the sample, including the interference signals caused by cross-reactivity of the recognition elements and those resulting from non-specific adsorption of matrix molecules in the sample solution to the sensing surface/interface. This challenge is motivating an increasing research effort to design a smart assay protocol, to discover new probe molecules with higher affinity and specificity, and to develop appropriate sample pre-treatment and antifouling strategies to diminish or prevent non-specific adsorption of coexisting molecules [149,151,152]. The latter is also important for preventing non-specific adsorption of the imaging tags in label-based assays. For example, a dual-antigen co-localization sensing scheme was employed in the specific detection of tumor-derived EVs to avoid false positives arising from binding of EVs released by normal somatic cells to the sensing surface [149]. On the other hand, although it is currently difficult to observe single fluorescent molecules or small NPs (e.g., QDs) with commercial wide-field microscopes, it is feasible to acquire scattering images of individual plasmonic NPs on a normal dark-field microscope, even using a consumer-grade digital camera as the photon detector [153]. With the continuous development of optical imaging setups toward miniaturization and standardization, we foresee that the scope of OSPIs in basic biological research studies and clinical applications will be further expanded. The second ongoing concern is the volume mismatch between existing nanotags and the molecules to be labeled. Compared with biomolecules with a diameter of a few nanometers (for example, antibody), available imaging nanolabels, such as QDs, SERS nanotags, and UCNPs, have diameters ranging from tens of nanometers to hundreds of nanometers. Such a large size mismatch would lead to two negative effects: decreased labeling efficiency due to steric hindrance and nonspecific adsorption due to gravity. Future work will focus on further development of high-quality imaging nanolabels that are similar in size to proteins and that are stable and bright enough. This can be accomplished either by enhancing the luminescence of existing small but less bright nanolabels with new physical principles (e.g., strong coupling in nanocavity) [154], or via construction of novel optical nanotags fulfilling the above criteria using cutting-edge techniques such as DNA origami [155]. Last but not least, although OSPI-based methodologies generally provide an assay sensitivity beyond picomolar levels, one should acknowledge that, in most cases, they still use similar or identical experimental principles and procedures to traditional ensemble analysis. For example, a multi-step on-chip hybridization, labeling and washing workflow has been adopted for OSPI-based nucleic acid detection [100]. The relatively slow mass transfer kinetics at solid–liquid interfaces, especially at the low molecular concentration levels, severely compromise the binding of target molecules and nanolabels to the sensing surface, and result in a long turnaround time for single testing. The integration of OSPIs with microfluids offers an opportunity to accelerate the interface reaction process and hence shorten the total assay time by leveraging the spatial confinement effect [156]. In addition, the use of biofunctionalized beads for target capture and labeling in homogeneous solutions before optical imaging of the individual nanolabels can also speed up the overall assay process and improve the detection throughput [157,158]. Moreover, we are witnessing the raid fusion of machine learning algorithms and bioanalytical techniques including the OSPIs-based methods [159,160,161], which not only facilitates automation of the data analysis, but also improves the accuracy of the assay results by enhancing the discrimination of measurement signals from the background noise. Taken together, with the fabrication of new nano-labels, combined with the rapid progress of automated demodulation of OSPI signals, we believe that OSPI analysis will serve as a promising tool for the detection of low abundance targets in biomedical scenarios.

## Figures and Tables

**Table 1:** Summary of the representative studies for OSPI.

Methods	Probes	Targets	Linear Range	Detection Limit	Ref.
Fluorescence	Cy3, Cy5	miRNA-21, miRNA-122	10 fM–1 nM, 10 fM–1 nM	5 fM, 5 fM	[83]
Fluorescence	QDs, Cy5	miRNA-21	1 fM–1 pM	1 fM	[95]
Fluorescence	QDs	DNA MTase	0.001–1 U/mL	0.0005 U/mL	[99]
Fluorescence	QDs	miRNA-375	/	10 aM	[100]
Fluorescence	UCNPs	PSA ^1^	0–500 pM	1 pM	[103]
Fluorescence	UCNPs	Aflatoxin B1	3.31–125 ng/mL	0.17 ng/mL	[104]
Fluorescence	UCNPs	PSA	0.1–1000 pg/mL	23 fg/mL	[105]
ECL	RuDSNS/ AuNPs	CK19 ^2^	0.01–10 ng/mL	0.12 pg/mL	[117]
SERS	Labeled AuNPs	miRNA-21	0.1–100 pM	0.1 pM	[131]
DFM	AuNPs	SrtA ^3^	0.05–50 nM	7.9 pM	[141]
DFM	AuNPs	IL-6 ^4^	/	7 pg/mL	[143]
DFM	AuNPs	miRNA-21	10–200 pM	2 pM	[144]
DFM	AuNRs, AuNPs	miRNA-21	0.02–1 nM	2 pM	[146]

^1^ Prostate-specific antigen, ^2^ Cytokeratin 19, ^3^ Sortase A, ^4^ Interleukin 6.

## Data Availability

Not applicable.
